# Attempts to prepare an all-carbon indigoid system

**DOI:** 10.3762/bjoc.11.42

**Published:** 2015-03-18

**Authors:** Şeref Yildizhan, Henning Hopf, Peter G Jones

**Affiliations:** 1 Institut für Organische Chemie, Technische Universität Braunschweig, Hagenring 30, D-38106 Braunschweig, Germany, Fax: (+49)531-391-5388; 2 Institut für Anorganische und Analytische Chemie, Technische Universität Braunschweig, Postfach 3329, D-38106 Braunschweig, Germany, Fax: (+49)531-391-5387

**Keywords:** α-methylene ketones, Cope rearrangement, cross-conjugation, indigo, McMurry coupling

## Abstract

First attempts are described to prepare a precursor for an all-carbon analog of indigo, the tetracyclic triene **4**. Starting from indan-2-one (**9**) the α-methylene ketone **13** was prepared. Upon subjecting this compound to a McMurry coupling reaction, it dimerized to the bis-indene derivative **17**, rather than providing the tetramethyl derivative of **4**, the hydrocarbon **14**. In a second approach, indan-1-one (**18**) was dimerized to the conjugated enedione **21** through the bis-1-indene dimer **19**. All attempts to methylenate **21** failed, however. When **19** was treated with the Tebbe reagent, the dimer **23** was produced, presumably through a Cope reaction of the intermediately generated isomer **22**. The bis-indene derivative **23** can be alkylated with 1,2-dibromoethane to produce a 1:1 mixture of the spiro compounds **24** and **25**. Although **9** could be reductively dimerized to **30**, the conversion of this olefin to **14** failed.

## Introduction

Cross-conjugated organic molecules are defined as unsaturated systems containing two π-electron systems (or lone pairs) that are in direct conjugation, whereby a third such system is excluded from interaction [3]. Typical examples are 2-vinyl-buta-1,3-diene ([3]dendralene, 3-methylene-penta-1,4-diene), benzophenone or urea.

Whereas the hydrocarbon parent systems, the [*n*]dendralenes, have long been a neglected class of oligoenes [4], the recent preparative accomplishments of the Sherburn group have changed the situation fundamentally [5–6]. These cross-conjugated hydrocarbons are now known up to [13]dendralene, and many of these potentially very valuable compounds are available in gram quantities, allowing, often for the first time, comprehensive chemical studies [6].

Notwithstanding modern progress, the phenomenon of cross-conjugation is as old as (scientific) organic chemistry. Many of the organic compounds that played an important role in the dawn of (industrial) organic chemistry are cross-conjugated systems or were converted into cross-conjugated organic salts during the color-generating process.

**Scheme 1:**
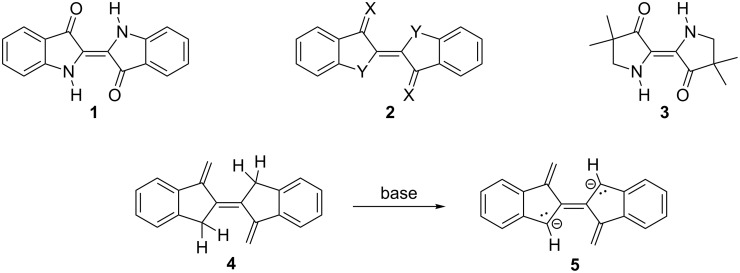
From indigo to heteroindigo derivatives and all-carbon-indigo.

This is illustrated by indigo (**1**, Scheme 1) and its derivatives (e.g., Tyrian purple) and the triphenylmethane-derived carbocations.

Starting from the generalized indigoid structure **2**, the role of the heteroatoms X and Y can be played not only by O and NH as in **1** itself, but by many other equivalent atoms and/or structural elements. In fact, the anellated benzene rings could be omitted altogether, resulting in what Lüttke, a pioneer in this area of dyestuff chemistry, has called urindigo (**3**, primordial indigo): representing the basic cross-conjugated π-electron system of the indigoid compounds [7–9].

Whereas the replacement of Y in **2** (X = O) by sulfur and selenium [10–11] or even tellurium [12–13] has been known for some time, derivatives of “phosphaindigo” (Y = various substituted phosphorus derivatives) have only been described more recently [14].

As far as we are aware, however, no attempt to prepare an all-carbon equivalent of indigo has ever been described. A system that could qualify as such an all-carbon analog of **1** is the bis-anion **5**, which itself should be obtainable by anionization of the linearly conjugated triene **4**.

Although we have to accept for the time being that our different approaches to preparing **4** (and **5**) have so far been unsuccessful (see below), we think that our initial efforts to attain this goal are worth publication. Furthermore, we are convinced that **5** will eventually become available.

## Results and Discussion

**Scheme 2:**
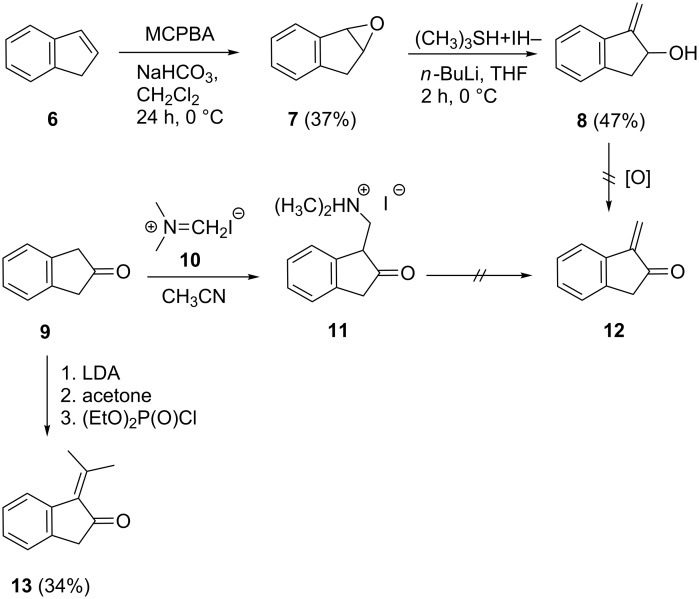
Attempts to prepare the α-methylene ketones **12** and **13**.

Our first attempt to prepare hydrocarbon **4** started from indene (**6**, Scheme 2). Epoxidation with *m*-chloroperbenzoic acid (MCPBA) according to a literature method [15] yielded the epoxide **7** in meager yields (Scheme 2).

The methylation of **7** to **8** was achieved by the treatment with trimethylsulfonium iodide in the presence of *n*-butyllithium in THF [16].

The spectroscopic data of these intermediates are incomplete in the chemical literature and are hence given in full detail in the experimental section (see Supporting Information File 1).

Unfortunately, all attempts to oxidize **8** to the α-methylene ketone **12**, failed (Dess–Martin reaction, IBX, Swern oxidation etc.); the original plan was to dimerize this intermediate to **4** by, e.g., a McMurry coupling reaction. Also the second route, starting with the reaction of 2-indanone (**9**) with the Eschenmoser salt **10** according to [17] to give the iodide **11**, was unsuccessful, since the attempted Hofmann elimination to **12** failed. Of course **12**, if formed, might not have survived the isolation process.

Since the target molecule **12** was expected to be a reactive compound, we decided to increase its stability by the introduction of two methyl substituents at its exocyclic double bond. Indeed, when **9** was first metalated with LDA and the resulting enolate quenched with acetone, the resulting ketol could be dehydrated in situ by treatment with diethyl chlorophosphate to yield **13** [18].

**Figure 1:**
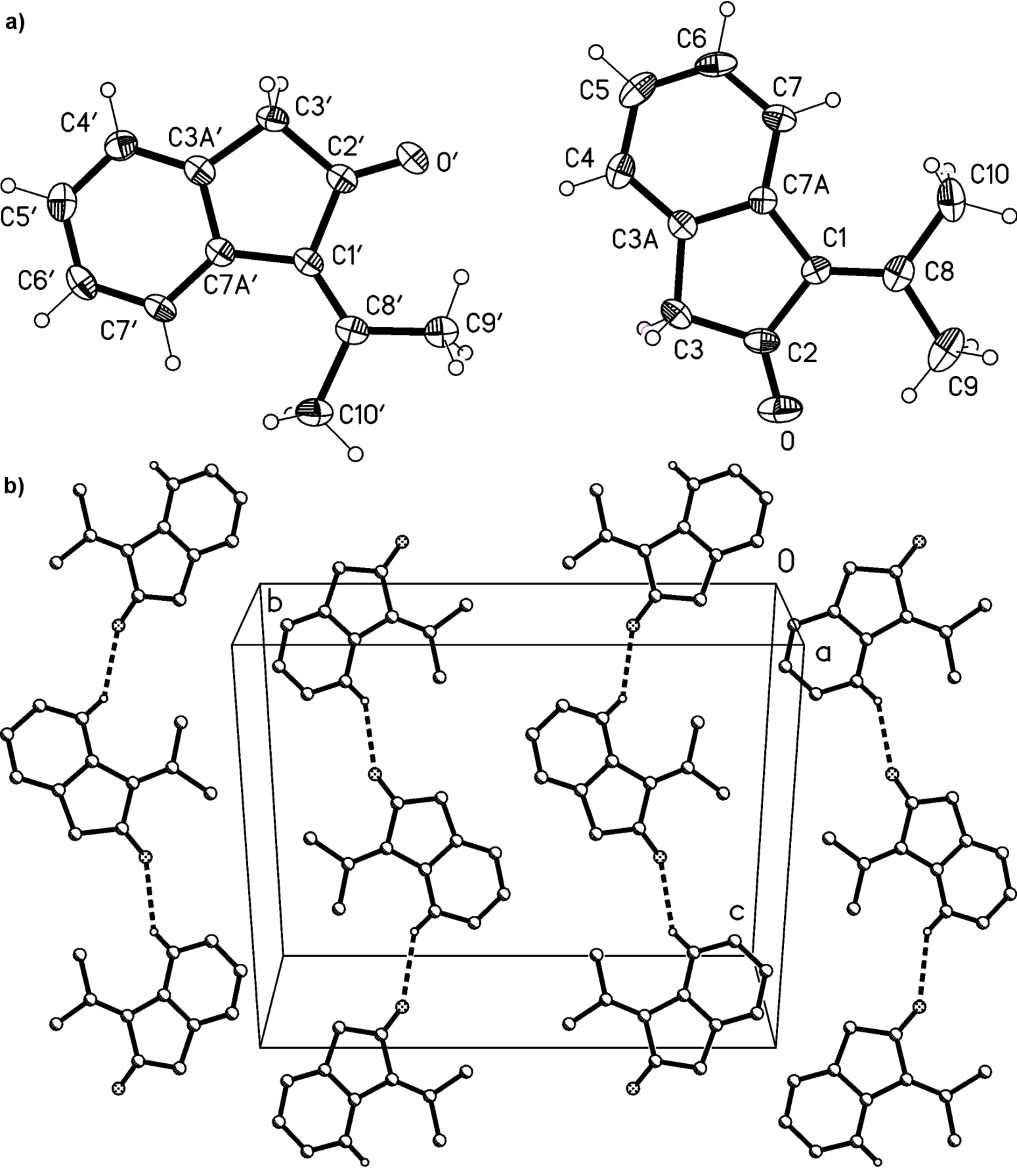
a) Both independent molecules of compound **13** in the crystal; ellipsoids represent 50% probability levels. b) Packing diagram of compound **13** (first independent molecule only) viewed perpendicular to (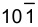). Dotted lines indicate the weak hydrogen bond H7···O, 2.50 Å, which links the molecules to form chains parallel to [101]. Other H atoms are omitted for clarity. The layers of the second molecule (not shown) are not topologically equivalent; they do not contain an equivalent interaction.

Derivative **13** is a crystalline solid that can be kept in the refrigerator for longer periods of time without decomposition. Slow evaporation of the solvent of a chloroform solution provided single crystals of **13** that were suitable for X-ray structure analysis. The resulting structure is shown in Figure 1.

Compound **13** crystallizes with two independent but closely similar molecules (rmsd 0.05 Å) in the asymmetric unit; these occupy independent alternating layers parallel to (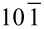). The ring systems are almost exactly planar (mean deviations <0.03 Å) with the atoms of the isopropylidene group lying approximately 0.2 Å to one side of the plane and the keto oxygen approximately 0.2 Å to the other side. The exocyclic C=C bonds are slightly lengthened, at 1.352(3), 1.353(3) Å.

**Scheme 3:**
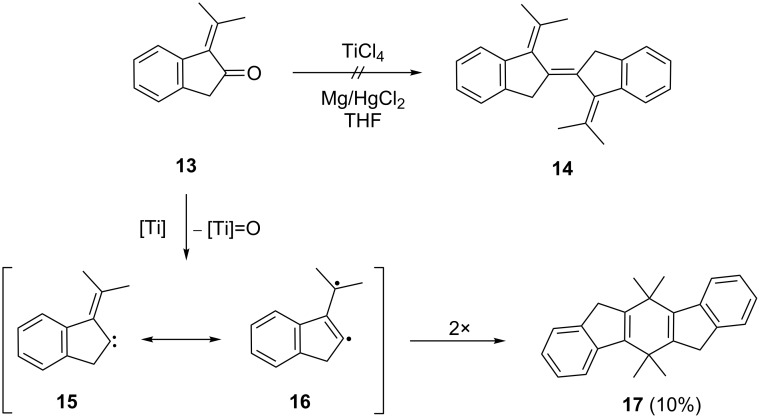
Dimerization of **13** under McMurry conditions.

Subjecting **13** to a McMurry coupling reaction (TiCl_4_/HgCl_2_ in THF) did not result in the formation of the expected dimer **14**, a tetramethyl derivative of **4**, but furnished dimer **17** in low yield (10%, Scheme 3).

**Figure 2:**
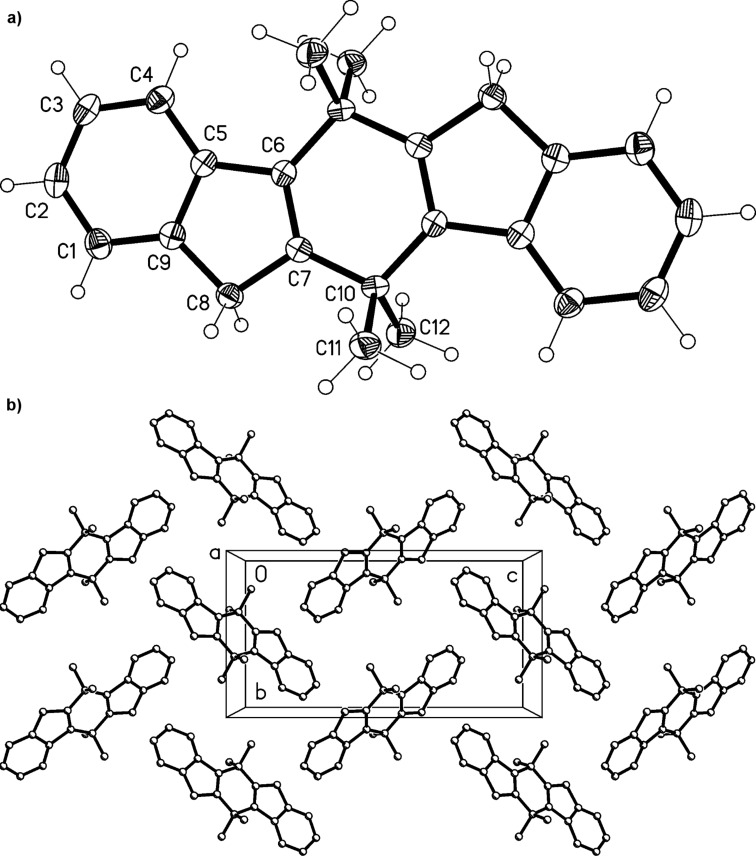
a) The molecule of compound **17** in the crystal; ellipsoids represent 50% probability levels. Only the asymmetric unit is numbered. b) Herringbone packing of compound **17** viewed parallel to the *a*-axis. H atoms are omitted.

To rationalize its formation we propose that the deoxygenation of **13** does indeed take place, but provides a carbenoid intermediate **15** rather than the vicinal diol complex that is usually postulated to be formed during the McMurry dimerization. Possibly the dimerization of the substrate molecule cannot take place because of steric hindrance by the *gem*-dimethyl group. Intermediate **15** is a vinylcarbene that, in principle, has several options to react further. It could dimerize to the intended product **14** (or its diastereomer), cyclize to a cyclopropene derivative or react via its resonance structure **16** to the isolated dimer **17**. Clearly, among these alternatives, the last route is preferred. In **17** the two benzene rings are anellated in *anti*-orientation, i.e., as far apart as possible. There exists an alternative structure, however, in which the two aromatic rings point in the same direction. To distinguish between these two possibilities based on spectroscopic evidence alone would not be easy. Fortunately, however, single crystals of the isolated dimer, suitable for X-ray analysis, were obtained, showing unambiguously that the *anti*-orientation is preferred (Figure 2).

The molecule of **17** exhibits crystallographic inversion symmetry, but the true symmetry is close to *C*_2_*_h_* (rmsd 0.02 Å). The ring system is planar, with a mean deviation of only 0.02 Å. The molecular packing involves a herringbone pattern in layers perpendicular to the *a*-axis. There are no noticeably short intermolecular interactions.

**Scheme 4:**
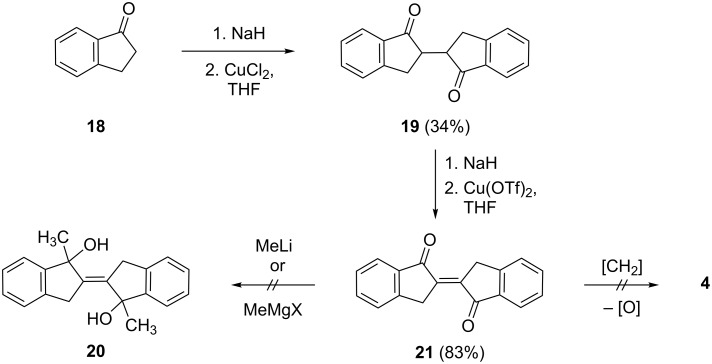
Dimerization of indan-1-one (**18**) by a stepwise approach.

Our next approach towards **4** (or a derivative) started from indan-1-one (**18**, Scheme 4).

Enolization of **18** was carried out with sodium hydride in THF and on subsequent oxidative dimerization the expected bis-ketone **19** was obtained. This compound is produced as a mixture of two diastereomers in roughly 1:1 ratio (NMR analysis), which can be separated by column chromatography. Since, however, in the next step the connecting single bond is transformed into a double bond, we used the diastereomeric mixture for the subsequent oxidation. This was carried out by metalating **19** with sodium hydride again, and then oxidizing the presumably resulting carbanion to **21** (a known compound [19]) with Cu(OTf)_2_, which gave cleaner results than the chloride employed previously.

Unfortunately, all efforts to convert **21** into **4** failed. Thus neither the Wittig reaction of **21** (MeP(Ph)_3_Br/*n*-BuLi, THF) nor its treatment with Oshima–Lombardo reagent (Zn, CH_2_Br_2_, TiCl_4_) [20–21] yielded a trace of **4**.

Likewise, the exposure of **21** to Tebbe’s reagent (trimethylaluminum with titanocene dichloride, pyridine, toluene) led to no sign of reaction in the desired sense. Nor was the tetramethyl derivative of **4**, hydrocarbon **14**, obtained when **21** was subjected to a crossed McMurry coupling with excess acetone. In a stepwise approach, **21** was treated with either methyllithium in ether or methylmagnesium bromide in the hope of either preparing a mono- or the bis-tertiary alcohol derivative **20**, which subsequently could be subjected to a dehydration reaction. In both cases only minute amounts of products could be obtained. When trying to purify these by column chromatography on silica gel, the stationary phase turned blue, but no defined products could be isolated.

**Scheme 5:**
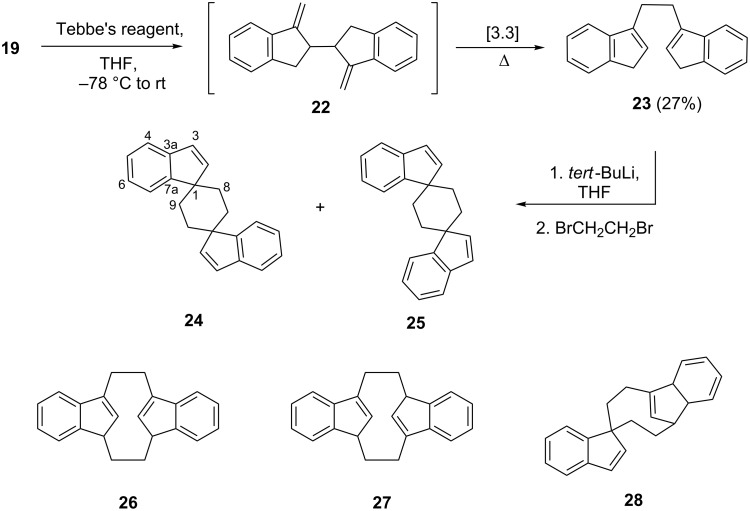
Methylenation of **19** and bisalkylation of the product **23** with 1,2-dibromoethane.

Since it might have been the central conjugated butenedione core that caused all these preparative difficulties, we next decided to investigate the behavior of diketone **19**, in which this conjugation is interrupted. Although its methylenation under Wittig conditions was again unsuccessful, the reaction with the Tebbe’s reagent provided a product. This, however, was not the hoped-for diene **22**, but an isomer, the hydrocarbon **23** (Scheme 5).

**Figure 3:**
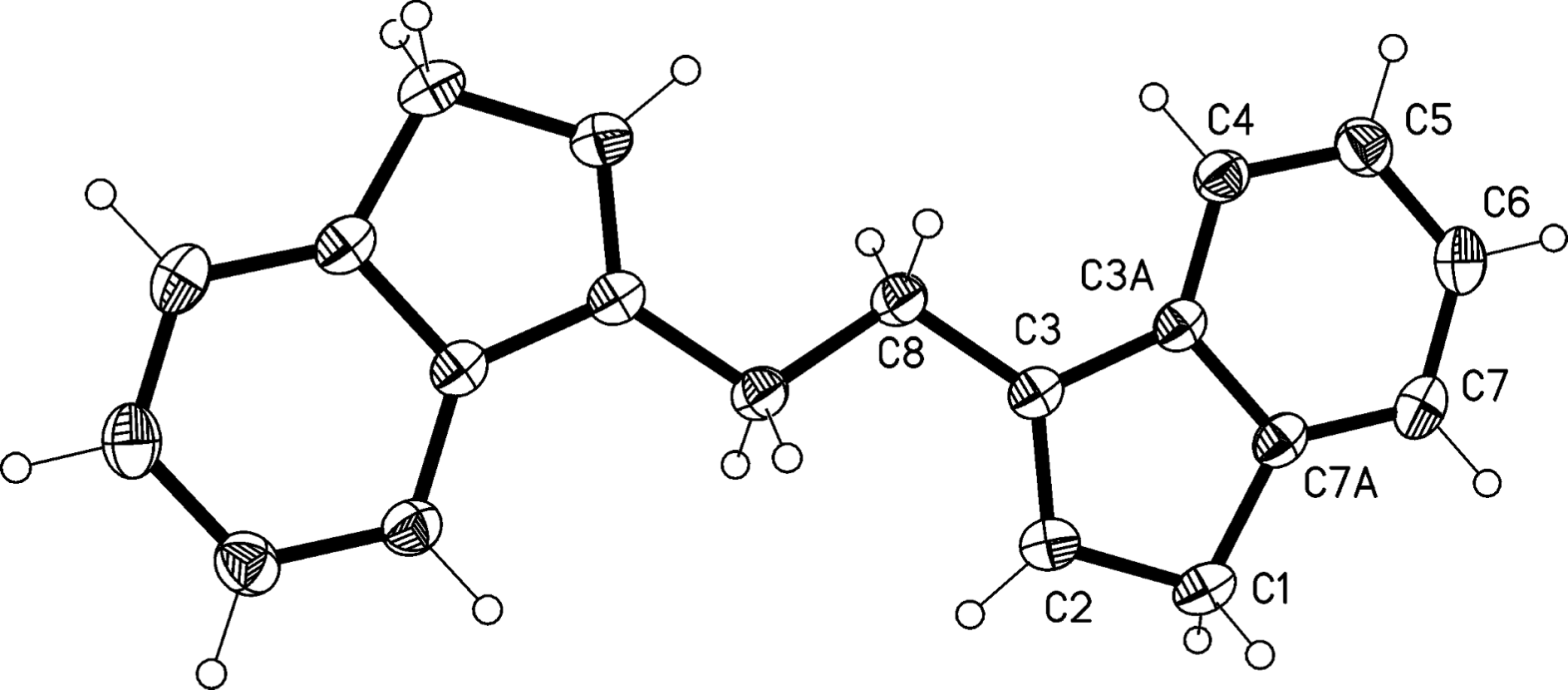
The molecule of compound **23** in the crystal. Ellipsoids represent 50% probability levels. Only the asymmetric unit is numbered.

The structure of hydrocarbon **23** was established by the usual spectroscopic data (see experimental section) and also by an X-ray crystal structure determination (Figure 3).

The molecule of **23** is planar (mean deviation 0.03 Å) and displays crystallographic inversion symmetry, although the true symmetry is close to *C*_2_*_h_* (rmsd 0.03 Å). The central C–C bond seems slightly short at 1.520(2) Å, but a similar value of 1.523(5) Å was observed in the only other known system with two analogous five-membered rings joined by a –CH_2_–CH_2_– moiety [22–23]. The molecular packing is devoid of striking features.

As far as the mode of formation of **23** is concerned, the isomerization formally is a [3.3]sigmatropic rearrangement (Cope rearrangement). Since the rearrangement of structurally similar compounds [24], including the parent system hexa-1,5-diene, requires much higher temperatures than those given in Scheme 5, we assume that the metalorganic reagent (or products derived therefrom) play a role in the isomerization.

Hydrocarbon **23** is, in fact, a known compound. It has been prepared previously from indene (**6**) by other routes [25–26], but always in the form of mixtures also containing other isomers. As far as we are aware, the above route is the only one that provides isomerically pure **23**, not surprising in view of its route of formation. These isomeric hydrocarbons are useful ligands for the preparation of bridged Ziegler–Natta catalysts employed for olefin polymerization [26–27].

Since compound **23** contains doubly activated methylene positions, it should be easy to alkylate or bis-alkylate it. Furthermore, use of a bis-electrophile such as 1,2-dibromoethane could lead to the [2.2]indenophane **26** or its isomer **27**, both potential ligands for the preparation of novel metallocene derivatives and also so far unknown.

The other conceivable spiro isomer **28** seems to be a less likely product since its *E*-configurated double bond (within a seven-membered ring) should result in considerable strain (anti-Bredt hydrocarbon).

**Figure 4:**
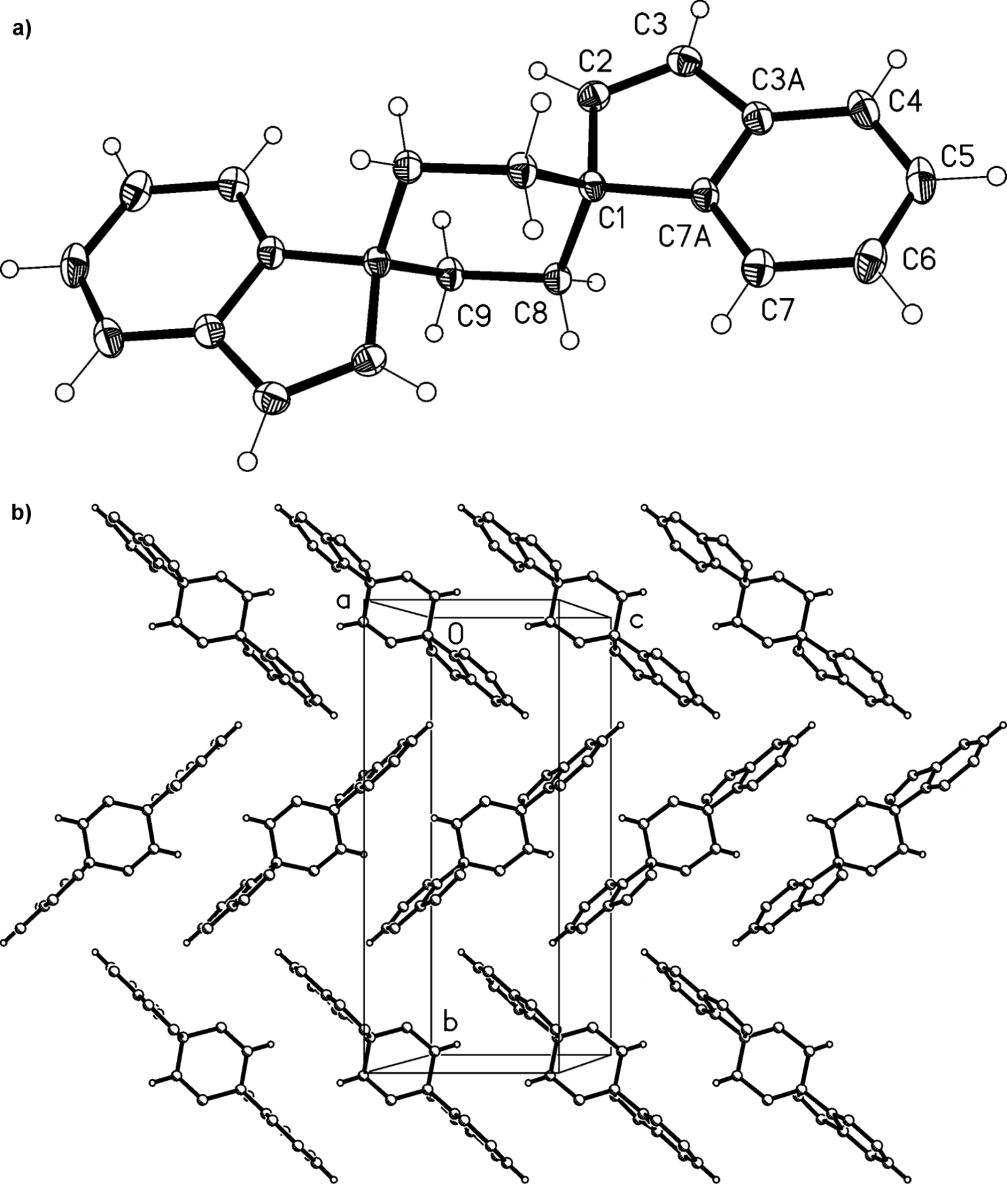
a) The molecule of compound **24** in the crystal. Ellipsoids represent 50% probability levels. Only the asymmetric unit is numbered. b) Packing diagram of compound **24** viewed perpendicular to the *bc* plane. C–H···π contacts to the centroid of the aromatic six-membered ring (seen side-on) can be recognized, but are not drawn explicitly. H atoms not involved in these contacts are omitted.

**Figure 5:**
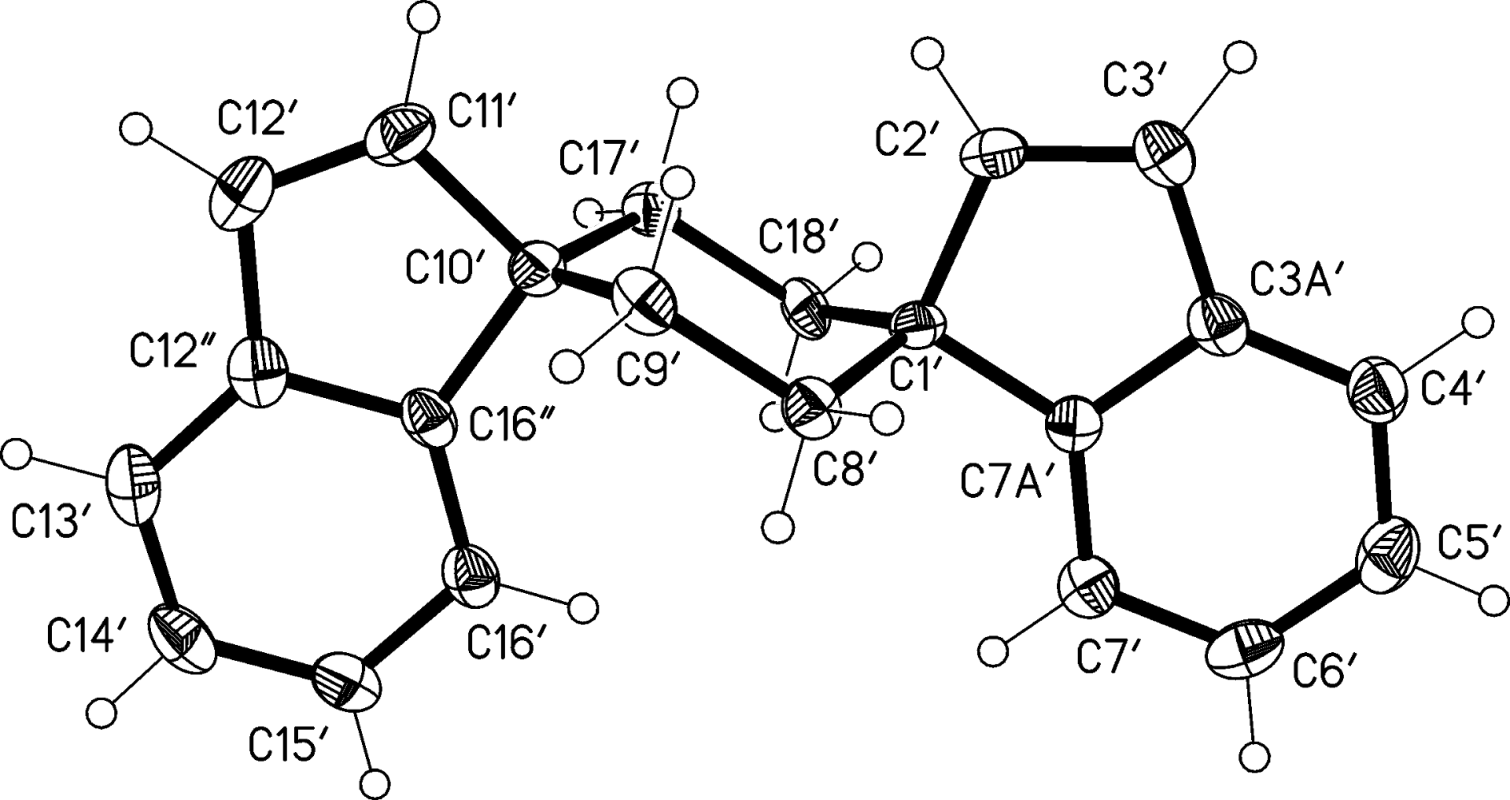
One of the two independent molecules of compound **25** in the crystal. Ellipsoids represent 50% probability levels.

When **23** was treated with *tert*-BuLi in THF and the presumably resulting bis-anion quenched with 1,2-dibromoethane, a complex product mixture consisting largely of polymeric material was formed. From this mixture, however, trace amounts of two hydrocarbons (total yield 5%) could be separated by thin layer chromatography. Further (column) chromatographic attempts to obtain preparative amounts of the two isomers in analytically pure form failed, however. The spectroscopic and analytical data of this hydrocarbon mixture (see experimental part) agreed with the two structural proposals **24** and **25** shown in Scheme 5. An unambiguous structure determination had to await X-ray crystallographic analysis. The required single crystals were obtained from the different chromatographic fractions (which contained either isomer in enriched form only) by recrystallization from chloroform and dichloromethane/chloroform, respectively. As can be seen from the X-ray structures, the former is the *anti*-spiro compound **24** (Figure 4) and the latter its *syn*-isomer **25** (Figure 5), with the former possessing the longer chromatographic retention time. Unfortunately, because of lack of material, no high quality NMR spectra of these two hydrocarbons could be obtained.

The molecule **24** displays crystallographic inversion symmetry, although the true symmetry is close to *C*_2_*_h_* (rmsd 0.02 Å). The molecular packing involves herringbone layers, parallel to the *bc* plane, in which two C–H···π contacts to the centroid of the aromatic six-membered ring are observed (H5···π 2.76 Å, H9A···π 2.83 Å). The structure determination of **25** was of limited accuracy because of twinning and disorder problems (indeed, there may be a small amount of contamination by **24**) and we therefore do not discuss it in detail. Both independent molecules display non-crystallographic mirror symmetry (rmsd 0.04 Å).

**Scheme 6:**
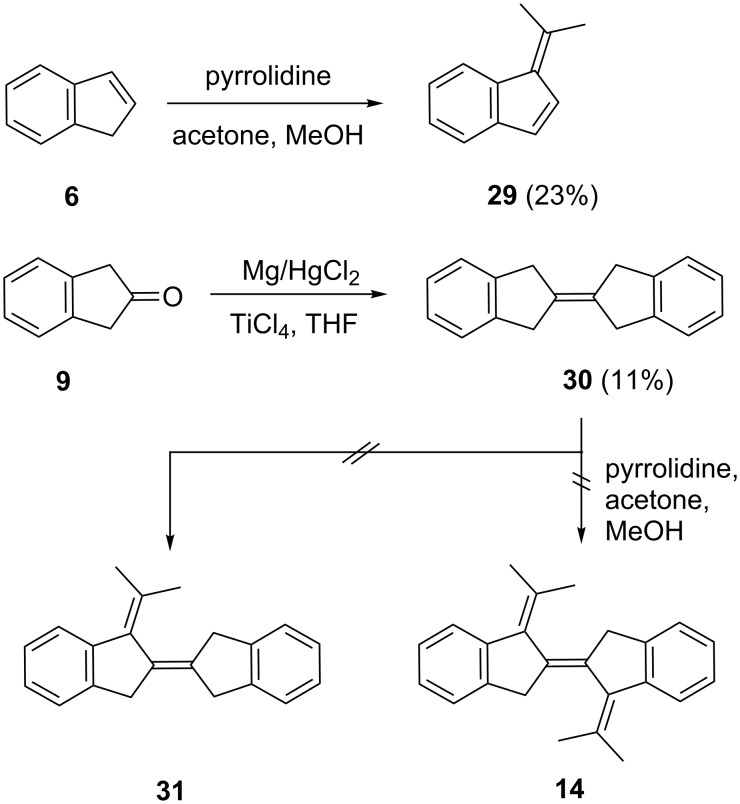
Cross-conjugated hydrocarbons by Thiele condensation.

Our final attempt for the present to prepare a precursor hydrocarbon for an all-carbon indigoid system rests on the observation that the Thiele condensation of indene (**6**) with acetone in pyrrolidine/methanol yields the benzofulvene **29** (Scheme 6).

**Figure 6:**
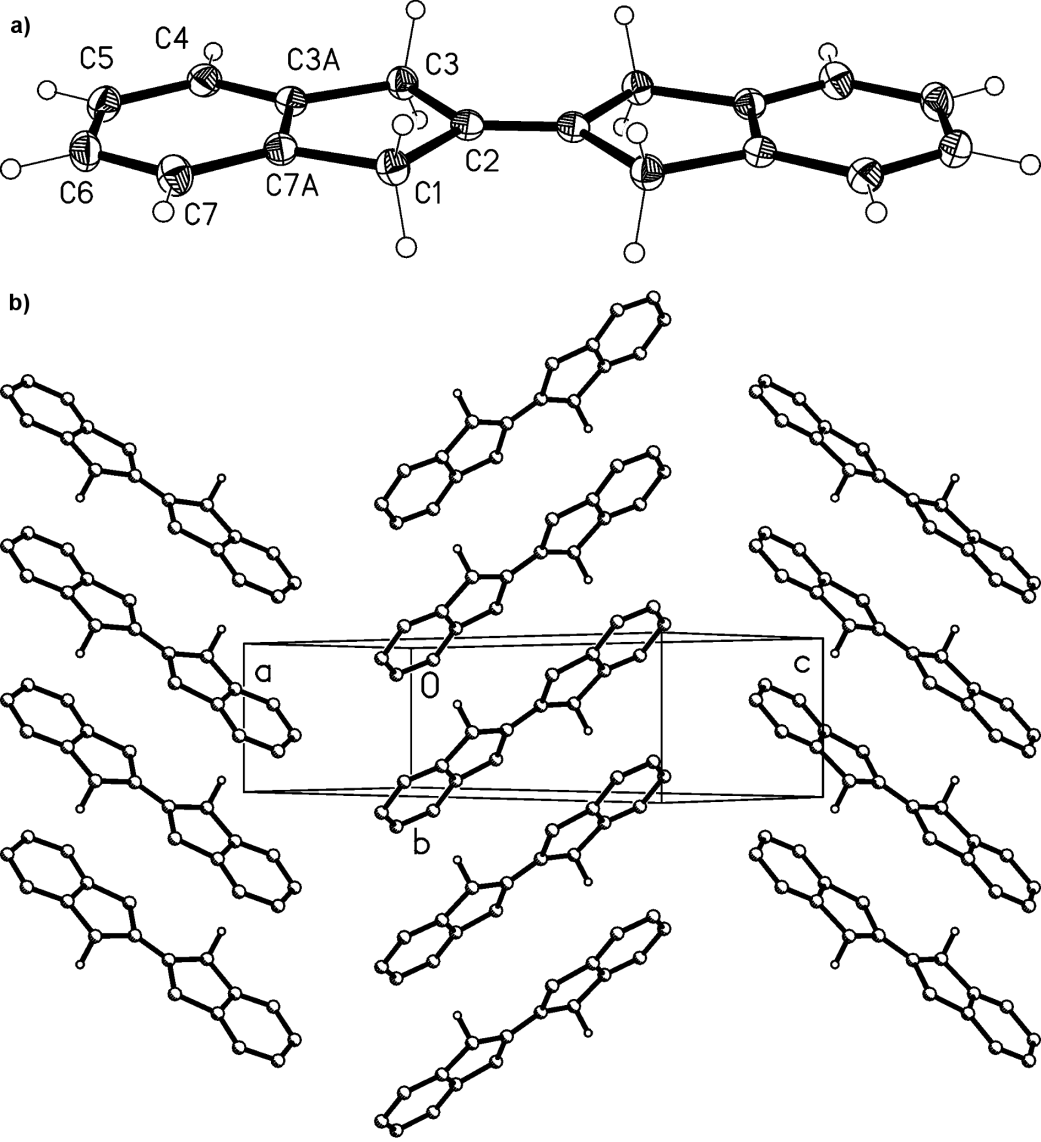
a) The molecule of compound **30** in the crystal. Ellipsoids represent 50% probability levels. Only the asymmetric unit is numbered. b) Packing diagram of compound **30** viewed perpendicular to (102). C–H···π contacts to the centroid of the six-membered ring can be recognized, but are not drawn explicitly. H atoms not involved in these contacts are omitted.

A suitable methylene component for this type of condensation would be the tetra-substituted olefin 2-(2-indanylidene)indane (**30**), in which each methylene group is activated both by a benzene ring and a double bond. We prepared this known olefin [28] by a McMurry-type dimerization of 2-indanone (**9**). The structure determination by the usual spectroscopic methods was straightforward and the obtained spectra agreed with the reported data [28]. The assignment was corroborated by a single-crystal X-ray structure determination (Figure 6); single crystals were obtained by slow evaporation of a pentane solution.

The molecule of **30** is planar (mean deviation 0.02 Å), but the true symmetry is close to *D*_2_*_h_* (rmsd 0.02 Å). The central C=C bond length is 1.327(2) Å. The molecular packing involves herringbone layers, parallel to the plane (102), in which C-H···π contacts from a methylene hydrogen to the centroid of the six-membered ring are observed (H1B···π 2.63 Å).

Unfortunately, all our condensation experiments with **30**, under conditions that were successful for **6**, failed and neither did we obtain the mono- (**31**) nor the (desired) bis-condensation product **14**.

## Conclusion

The preparation of a suitable precursor for the first all-carbon analog of an indigoid dyestuff has failed so far. It is conceivable that unsubstituted α-methylene ketones such as **12** are too reactive to be isolated under the reaction conditions. On the other hand, if the methylene group is protected by two methyl groups, the corresponding ketone, **13**, becomes isolable, but fails to participate in a McMurry dimerization, possibly because of steric hindrance. If the double bond between the indane-derived “halves” in, e.g., **4** is replaced by a single bond (as in **22**), a Cope reaction is the most favorable process yielding the ethano-bridged bis-indenyl derivative **23**, which can be employed for the introduction of additional linker units, but is of no use for the preparation of the intended target molecule **4**.

## Supporting Information

File 1 Characterization data.
