# Supplementary material for: Attempts to prepare an all-carbon indigoid system
Source: Beilstein J Org Chem. 2015 Mar 18;11:363–72. doi: 10.3762/bjoc.11.42 (PMC4419558; doi:10.3762/bjoc.11.42)
Supplement: File 1 — Characterization data. [file Beilstein_J_Org_Chem-11-363-s001.pdf]

Supporting Information

for

Attempts to prepare an all-carbon indigoid system

Şeref Yildizhan[1], Henning Hopf[1*], and Peter G. Jones[2]

Address: [1]Institut für Organische Chemie, Technische Universität Braunschweig, Hagenring 30, D-38106 Braunschweig, Germany, Fax: (+49)531-391-5388 and [2]Institut für Anorganische und Analytische Chemie, Technische Universität Braunschweig, Postfach 3329, D-38106 Braunschweig, Germany, Fax: (+49)531-391-5387

E-mail: Henning Hopf - h.hopf@tu-bs.de

*Corresponding author

Alicyclic Compounds, Part IX. [1]; for Part VIII see [2]. Dedicated to Professor Wolfgang Lüttke on the occasion of his 95th birthday

Characterization data

General remarks:

Chromatography: TLC: Polygram Sil G/$UV_{254,}$ Macherey, Nagel & Co. (Düren). – CC: Kieselgel 60 (70-230 mesh), Merck. – M. p.: < 200 °C: Büchi 510, > 200 °C: Kofler "Thermopan", Reichert (Vienna); the m.p.s are uncorrected. – **NMR**: ^{1}H- and ^{13}C-spectra: Bruker AC-200, ^{1}H NMR: 200.1 MHz), ^{13}C NMR: 50.3 MHz; Bruker DRX-400, ^{1}H NMR: 400.1 MHz), ^{13}C NMR: 100.6 MHz; int. standard: TMS; spin multiplicities of ^{13}C signals: DEPT-technique. – **IR**: solids: KBr pellets, liquids: film, NICOLET 320 FT-IR-spectrometer; ATR-IR-spectra: Bruker Tensor 27. - **UV/Vis**: HP 8452A Diode Array or Varian Cary 100 BIO. – **MS**: FINNIGAN MAT 90x, EI: 70 eV or CI (ammonia). **GC/MS**: FINNIGAN TSQ 700 (EI, 70 eV) coupled to a HP 5890A GC.

1,2-Epoxyindane (7): To freshly distilled indene (**6**, 11.6 g, 0.1 mol) in dichloromethane (50 mL) was added at 0 °C a solution of *m*-chloroperbenzoic acid (2.0 g, 11.6 mmol) in dichloromethane (50 mL). The reaction mixture was stored in a refrigerator overnight and then washed with a sat. aqueous bicarbonate solution. After washing the aqueous phase twice with dichloromethane, the organic phases were combined and dried ($MgSO_4$). The solvent was removed in vacuo and the crude product purified by column chromatography (silica gel; hexane/ethyl acetate = 95:5): 4.9 g (37%) of **6** as a colorless oil. - **^{1}H NMR** (400 MHz, $CDCl_3$): δ = 2.98 (dd, J_1 = 3, J_2 = 18

Hz, 1 H, 3-H), 3.24 (d, *J* = 18 Hz, 1 H, 3-H), 4.15 (t, *J* = 3 Hz, 1 H, 2-H), 4.31 (br. d, *J* = 3 Hz, 1 H, 1-H), 7.25-7.36 (m, 3 H, 4-, 5-, 6-H), 7.56 ppm (d, *J* = 7.3 Hz, 1 H, 7-H). - **^{13}C NMR** (100 MHz, $CDCl_3$): δ = 34.2 (t, C-3), 57.2 (d, C-2), 58.6 (d, C-1), 124.8, 125.7, 125.8, 128.1 (4 x d, C-4, -5, -6, -7), 140.6, 143.3 ppm (2 x s, C-3a, -7a. - **IR** (diamond-ATR): nu(tilde) = 3491 (w), 3585 (w), 3093 (w), 3072 (m), 3045 (m), 3029 (m), 1752 (m), 1726 (m), 1475 (m), 1465 (m), 1419 (m), 1372 (m), 1232 (m), 1227 (m), 1203 (m), 1175 (m), 983 (m), 903 (m), 829 cm^{-1} (s). - **UV/Vis** (CH_3CN): λ_{max} (lg ε) = 198 (4.4), 212 (3.9), 224 (3.7), 230 (3.59), 268 nm (2.9). - **MS** (EI, 70 eV): *m/z* (%) = 132 (59) [M^+], 104 (100), 78 (32), 51 (20). – The compound has been described in the literature [3]; the spectroscopic data reported here are more up-to-date and more comprehensive.

1-Methylenindan-2-ol (8): To a suspension of trimethyl-sulfonium iodide (13.9 g, 68.1 mmol) in anhydrous THF (200 mL) was added at -78 °C *n*-butyl lithium (42.5 mL, 68.0 mmol of a 1.6 M solution in hexane). The reaction mixture was slowly warmed to 0 °C and a solution of **7** (3.0 g, 22.7 mmol) in THF (20 mL) was added drop-wise. The reaction mixture was brought to room temp. overnight and hydrolyzed with 100 mL of water. The THF was removed by rotary evaporation and the remainder taken up in diethyl ether (200 mL). The organic phase was washed with water and dried with magnesium sulfate. The

solvent was removed in vacuo and the crude material purified by column chromatography on silica gel using pentane with increasing amounts of diethyl ether: 1.55 g (47%) of **8**, colorless solid, m. p. 61 °C. The compound has been described in the literature [4]; the spectroscopic data reported here are more up-to-date and more comprehensive. - **^{1}H NMR** (400 MHz, $CDCl_3$): δ = 1.92 (s, 1 H, OH), 2.90 (dd, 1 H, J_1 = 4.1, J_2 = 16.6 Hz, 3-H), 3.34 (dd, 1 H, J_1 = 7.4, J_2 = 16.6 Hz, 3-H), 4.98 (d, 1 H, J = 4.5 Hz, 2-H), 5.37 (d, 1 H, J = 1.8 Hz, 8-H), 5.65 (d, 1 H, J = 2.1 Hz, 8-H), 7.25-7.32 (m, 3 H, 4-, 5-, 6-H), 7.53 ppm (d, J = 6.3 Hz, 1 H, 7-H). - **^{13}C NMR** (100 MHz, $CDCl_3$): δ = 40.6 (t, C-3), 74.4 (d, C-2), 105.6 (t, C-8), 121.2 (d, C-7), 125.5, 127.0, 129.1 (3 x d, C-4, -5, -6), 138.5 (s, C-7a), 142.5 (s, C-3a), 153.3 ppm (s, C-1). - **IR** (KBr): nu(tilde) = 3279 (s), 3356 (s), 3068 (m), 3039 (m), 2945 (m), 2924 (m), 2906 (m), 2871 (m), 1473 (m), 1461 (m), 1424 (m), 1321 (s), 1205 (m), 1118 (m), 1046 (s), 1019 (m), 893 (s), 780 (s), 736 (s), 695 (m), 622 cm^{-1} (m). - **UV/Vis** (CH_3CN): λ_{max} (lg ε) = 210 (4.3), 252 (4.1), 290 (3.5), 302 nm (3.4). - **MS** (EI, 70 eV): *m/z* (%) = 146 (100) [M^+], 147 (49), 131 (28), 129 (13), 128 (30), 127 (19), 117 (65), 116 (48), 115 (83), 91 (13).

1-Isopropylideneindan-2-one (13): To a solution of LDA (24 mL, 48 mmol; commercial solution in THF/heptane/ethylbenzene) was added at -78 °C 2-indanone (**9**; 6.0 g, 45.5 mmol) in THF (20 mL). After stirring the mixture for 1 h at -78 °C, acetone (34

mL, 0.455 mol) was added. Stirring was continued for an additional hour at -78 °C, diethyl chlorophosphate (6.9 mL, 47.7 mmol) was added, and the reaction mixture brought to room temp. overnight. For work-up water was added (50 mL) and the THF removed by rotary evaporation. The residue was taken up in diethyl ether (500 mL), the organic phase was washed with water, and dried ($MgSO_4$). Most of the ether was removed in vacuo and the remaining solution purified by silica gel column chromatography (pentane : diethyl ether = 9 : 1): 2.63 g (34%) of **13**, colorless solid, m. p. 77 °C (decomp.) which slowly turned greenish on standing [5]. Single crystals suitable for X-ray structure determination were obtained by sublimation. - **^{1}H NMR** (400 MHz, $CDCl_3$): δ = 2.31, 2.50 (2 x s, 3 H each, 9-, 10-H), 3.49 (s, 2 H, 3-H), 7.22-7.25 (m, 1 H, 4-H), 7.25-7.28 (m, 1 H, 5-H), 7.28-7.33 (m, 1 H, 6-H), 7.61 ppm (d, *J* = 7.6 Hz, 1 H, 7-H). - **^{13}C NMR** (100 MHz, $CDCl_3$): δ = 23.5, 25.2 (2 x q, C-9,-10), 43.1 (t, C-3), 48.2 (t, C-8), 124.3 (d, C-7), 125.0 (d, C-6), 127.0 (d, C-5), 127.2 (d, C-4), 131.5 (d, C-8), 136.7 (d, C-1), 140.9 (d, C-7a), 149.9 ppm (d, C-3a). - **IR** (KBr): nu(tilde) = 3393 (w), 2966 (w), 1752 (m), 1701 (s), 1603 (m), 1574 (m), 1464 (m), 1388 (m), 1362 (m), 1187 (m), 1157 (m), 1090 (m), 1055 (m), 916 (w), 760 (s), 728 (s), 684 (m), 622 cm^{-1} (m). - **UV/Vis** (CH_3CN): λ_{max} (ε) = 200 (4.4), 234 (3.8), 274 (3.5), 306 nm (3.4). – **GC/MS** (EI, 70 eV): *m/z* (%) = 172 (69) [M^+], 157 (11), 144 (13), 129 (100), 128 (44), 127 (14), 115 (10).

5,5,11,11-Tetramethyl-5,6,11,12-tetrahydroindeno[1,2-*b*]fluorene (17): To a suspension of $HgCl_2$ (244 mg, 0.9 mmol) in THF (10 mL) was added magnesium (875 mg, 36 mmol, turnings) and the mixture was vigorously stirred under nitrogen. The mother liquor was removed (pipet) from the precipitate, which was subsequently washed (3 x 5 mL of THF). To this residue was added THF (20 mL), the mixture was subsequently cooled to -10 °C and $TiCl_4$ (2 mL, 18 mmol) was added dropwise. To the yellow/green reduction mixture was added a solution of **13** (770 mg, 4.5 mmol) in anhydrous THF (10 mL). After additional stirring for 2 h at 0 °C the reaction mixture was heated to reflux for 24 h. After cooling to room temp., diethyl ether (100 mL) was added, the solution filtered through celite, and the solvent removed in vacuo. The crude product mixture was purified by column chromatography (silica gel, pentane): 145 mg (10 %) of **17**, colorless solid, m. p. 325 °C. Single crystals suitable for X-ray structure determination were obtained by slow evaporation of a pentane solution. - **^{1}H NMR** (400 MHz, $CDCl_3$): δ = 1.64 (s, 12 H, 13-, 14-H), 3.49 (s, 4 H, 6-, 12-H), 7.12-7.19 (m, 2 H, 2-, 8-H), 7.26-7.33 (m, 2 H, 3-, 9-H), 7.47 (d, J = 7.3 Hz, 2 H, 4-, 10-H), 7.57 ppm (d, J = 7.3 Hz, 2 H, 1-, 7-H). - **^{13}C NMR** (100 MHz, $CDCl_3$): δ = 28.0 (q, C-13, -14), 35.4 (t, C-6, -12), 35.7 (s, C-5, -11), 121.2 (d, C-1, -7), 123.6 (d, C-2, -8), 123.9 (d, C-4, -10), 125.9 (d, C-3, -9), 139.6 (s, C-4b, -10b), 143.7 (s, C-4a, -10a), 143.9 (C-5a, -11a), 148.0 ppm (s, C-6a, -12a). - **IR** (diamond-ATR): nu(tilde)

= 3079 (w), 3057 (w), 2970 (m), 2925 (m), 2925 (m), 1594 (m), 1461 (m), 1381 (m), 1206 (m), 815 (w), 765 (s), 726 cm^{-1} (s). - **UV/Vis** (CH_3CN): λ_{max} (lg ε) = 193 (4.4), 208 (4.6), 258 nm (4.4). – **GC/MS** (EI, 70 eV): *m/z* (%) = 312 (18) [M^+], 298 (26), 297 (100), 283 (22), 282 (95), 268 (8), 267 (35), 266 (13), 265 (17), 252 (20), 141 (14), 133 (8), 132 (8), 126 (9). - **HRMS** ($C_{24}H_{24}$, 312.45): calcd.: 312.1878; found: 312.1869 ± 0.3 ppm. – **Element. anal.**: ($C_{24}H_{24}$, 312.45): calcd.: C 92.26, H 7.74; found C 91.82 H 7.76.

2,3,2´,3´-Tetrahydro-[2,2´]biindenyl-1,1´-dione (19): To a sus-pension of sodium hydride (2.1 g, 83.3 mmol) in anhydrous THF (150 mL) a solution of indan-1-one (**18**, 10.0 g, 75.8 mmol) in THF (250 mL) was added dropwise. The reaction mixture was stirred overnight, cooled to -78 °C, and $CuCl_2$ (11.2 g, 83.3 mmol) was added. After stirring for 30 min at this temperature, the mixture was brought to room temp. For work-up, water was added (40 mL), the THF was removed by rotary evaporation, and the residue taken up in diethyl ether (250 mL). The organic phase was dried ($MgSO_4$) and filtered through a pad of silica gel. After the solvent had been removed, the residue (3.4 g, 34%) was recrystallized from methanol: 911 mg of a yellow-green solid, m. p. 140 °C [6]. - **^{1}H NMR** (400 MHz, $CDCl_3$): δ = 2.47-2.98 (m, 2 H, 2-H), 3.18-3.58 (m, 4 H, 3-H), 7.38-7.81 ppm (m, 8 H, 4-, 5-, 6-, 7-H). - **^{13}C NMR** (100 MHz,

$CDCl_3$): δ = 28.2, 30.8 (2 x t, C-3), 47.0, 48.2 (2 x d, C-2), 123.9, 123.9 (2 x d, C-7), 126.4, 126.6 (2 x d, C-5), 127.5, 127.6 (2 x d, C-8), 134.5, 135.0 (2 x d, C-6), 136.8, 136.8 (2 x d, C-7a), 153.3, 153.8 (2 x d, C-3a), 206.2, 207.3 ppm (2 x s, C-1). - **IR** (KBr): nu(tilde) = 3423 (w), 3067 (w), 2943 (w), 1705 (s), 1699 (s), 1608 (m), 1586 (w), 1295 (w), 754 cm^{-1} (m). - **UV/Vis** (CH_3OH): λ_{max} [nm] (lg ε) = 212 nm (0.65). - **MS** (EI, 70 eV): *m/z* (%) = 262 (100) [M^+], 131 (78), 104 (35), 79 (86).

Enedione 21: According to the literature procedure [6], a suspension of sodium hydride (640 mg, 16 mmol) in THF (50 mL) was added dropwise to a solution of **19** (1.67 g, 6.40 mmol) in THF (50 mL). The reaction mixture was stirred overnight and then $Cu(OTf)_2$ (5.78 g, 16.0 mmol) was added in one portion. After stirring for 2 h, water (20 mL) was added, the solvent was removed in vacuo, and the resulting residue taken up in chloroform. After washing the organic phase with water, drying ($MgSO_4$) and solvent removal, the residue was recrystallized from methanol: 1.38 g (83%) of **21** as a yellow-green solid, m. p. > 225 °C (decomp.) - **^{1}H NMR** (400 MHz, $CDCl_3$): δ = 4.26 (s, 2 H, 3-H), 7.31-7.40 (m, 1 H, 6-H), 7.51 (d, *J* = 7.6 Hz, 1 H, 4-H), 7.56-7.60 (dd, J_1 = 1.1, J_2 = 7.7 Hz, 1 H, 6-H), 7.80 ppm (d, *J* = 7.7 Hz, 2 H, H-4). - **^{13}C NMR** (100 MHz, $CDCl_3$): δ = 32.3 (t, C-3), 124.1 (d, C-7), 126.6 (d, C-4), 127.6 (d, C-5), 135.4 (d, C-6), 138.2 (s, C-3a), 138.2 (s, C-7a), 151.0 (s, C-2), 196.9 (s, C-1). - **IR** (KBr): nu(tilde) = 3419 (w), 3071

(w), 1711 (s), 1686 (m), 1682 (m), 1605 (m), 1465 (w), 1276 (m), 749 cm^{-1} (w). - **UV/Vis** (CH_3OH): λ_{max} (lg ε) = 194 (4.5), 202 (4.6), 248 (4.1), 294 (3.8), 316 (3.6), 336 (3.3), 360 nm (3.0). - **MS** (EI, 70 eV): *m/z* (%) = 260 (100) [M^+], 231 (78), 215 (21), 202 (59), 132 (27), 101 (26).

1,2-Bis(1-indenyl)ethane (23): A solution of $Al(CH_3)_3$ (44 mL, 88.2 mmol of a commercial 2 M solution in toluene) was added to titanocene dichloride (10 g, 40.1 mmol). After stirring for 3 d at room temperature an additional portion of $Al(CH_3)_3$ was added (16 mL, 32 mmol) and the mixture was stirred for another 12 h. The metal-organic solution thus generated was added dropwise to a solution of **19** (1.88 g, 7.2 mmol) in anhydrous THF (50 mL) at -78 °C. The mixture was warmed to room temp. overnight, and water was added until the gas evolution ceased. The reaction mixture was filtered through a pad of silica, taken up in diethyl ether, and purified by column chromatography (silica gel, pentane) after most of the solvent had been removed. Recrystallization from diethyl ether provided 502 mg (27%) of **23** as a colorless solid, m. p. 190 °C. - Single crystals suitable for X-ray structure determination were obtained by slow evaporation of a diethyl ether solution. - **^{1}H NMR** (400 MHz, $CDCl_3$): δ = 3.05 (s, 2 H, 8-H), 3.44 (br. s, 2 H, 1-H), 6.38 (br. s, 1 H, 2-H), 7.19-7.23 (dd, J_1 = 1.1, J_2 = 7.4 Hz, 1 H, 5-H), 7.29-7.33 (dd, J_1 = 1.1 Hz, J_2 = 7.4 Hz, 1 H, 6-H), 7.40-7.42 (d, J = 7.4 Hz, 1 H, 4-

H), 7.46-7.49 ppm (d, J = 7.4 Hz, 1 H, 7-H). - **^{13}C NMR** (100 MHz, $CDCl_3$): δ = 26.4 (t, C-1), 37.8 (t, C-4), 118.9 (d, C-6), 123.8 (d, C-9), 124.6 (d, C-8), 126.1 (d, C-7), 128.0 (d, C-2), 144.2 (s, C-3a), 144.5 (s, 7a), 145.4 ppm (s, C-3). - **IR** (diamond-ATR): nu(tilde) = 3066 (w), 2896 (w), 1459 (m), 1394 (m), 1053 (w), 965 (m), 919 (m), 884 (m), 865 (m), 764 (s), 732 (s), 713 (s), 683 cm^{-1} (m). - **UV/Vis** (CH_3CN): λ_{max} (lg ε) = 206 (4.6), 252 (4.3), 282 (3.4), 292 (3.5), 304 nm (3.3). - **GC/MS** (EI, 70 eV): m/z (%) = 258 (100) [M^+], 130 (81), 129 (57), 128 (100), 127 (17), 115 (12). - $C_{20}H_{18}$ (258.36): calcd.: C 92.98 H 7.02, found C 92.99 H 7.05.

Spiro compounds 24 and 25: To a solution of **23** (554 mg, 2.1 mmol) in anhydrous THF (200 mL) was added at –78 °C within 30 min a solution of *tert*-BuLi (3.6 mL, 5.4 mmol of a 1.5 M solution in pentane). The reaction mixture was warmed to room temp., and stirred for another 4 h until a red-brown homogenous solution had been produced. After cooling to –78, °C a solution of 1,2-dibromoethane (0.19 mL, 2.1 mmol) in THF (10 mL) was added and the mixture allowed to warm up to room temp. overnight. Water (1 mL) was added and the THF was removed by rotary evaporation. The residue was taken up in diethyl ether and the solution washed with water, dried ($MgSO_4$), and filtered through a pad of silica gel. After solvent removal the residue was purified by column chromatography (silica gel, pentane).

Recrystallization from dichloromethane provided 21 mg (5%) of the two hydrocarbons **24** and **25** (ratio 1:1, ^{1}H NMR analysis). Single crystals of **24** and **25** suitable for X-ray structure determination were obtained by slow evaporation from deuteriochloroform and dichloromethane/deuteriochloroform solutions in N.M.R. tubes. - Spectroscopic data for hydrocarbon mixtures: **^{1}H NMR** (400 MHz, $CDCl_3$): δ = 1.82 (br. s, 2 H), 2.06 (br. s, 2 H), 6.72-6.76 (m, 2 H), 7.25-7.32 (m, 4 H), 7.34-7.37 (dd, J_1 = 1.9, J_2 = 7.2 Hz, 2 H), 7.77 ppm (d, J = 6.2 Hz, 2 H). - **^{13}C NMR** (100 MHz, $CDCl_3$): δ = 30.9 (t), 52.8 (s), 121.6 (d), 122.8 (d), 124.8 (d), 126.9 (d), 128.9 (d), 143.5 (s), 152.5 ppm (s). - **IR** (diamond-ATR): nu(tilde) = 3060 (w), 2853 (w), 1451 (m), 1367 (w), 1012 (w), 936 (m), 891 (w), 779 (m), 754 (s), 738 (s), 695 (m), 661 cm^{-1} (m). - **UV/Vis** (CH_3CN): λ_{max} (lg ε) = 211 (3.5), 217 (3.5), 254 nm (3.1). – **GC/MS** (EI, 70 eV): *m/z* (%) = 285 (20), 284 [M^+] (80), 257 (11), 256 (52), 255 (27), 156 (40), 155 (61), 130 (14), 129 (75), 128 (100), 127 (20), 115 (32). - **HRMS** ($C_{22}H_{20}$, 284.16): calcd. 284.1565¸ found 284.1556 ± 0.3 ppm. –

X-Ray structure determinations

All compounds crystallized solvent-free. Crystals were mounted in inert oil on glass fibres and transferred to the cold gas stream of the diffractometer (Bruker SMART 1000 CCD). Intensity data were recorded using monochromated Mo $K\alpha$ radiation (λ = 0.71073 Å). No absorption corrections were performed. The structures were refined anisotropically on F^2 using the program SHELXL-97 [7]. Hydrogen atoms were refined using rigid methyl groups allowed to rotate but not tip, or a riding model starting from calculated positions. The crystal of **25** was of poor quality (weak diffractor and also non-merohedrally twinned), and additionally one of the two molecules displays significant residual electron density (ca. 0.7 e Å^{-3}) and may be slightly disordered. The *R* values are correspondingly high. This structure should therefore be regarded only as a qualitative confirmation that the correct isomer has been formed.

Crystallographic data have been deposited with the Cambridge Crystallographic Data Centre as supplementary publications no. CCDC-1031347 (**13**), -1031348 (**17**), -1031349 (**23**), -1031350 (**24**), -1031351 (**25**), -1031352 (**30**). Copies of the data can be obtained free of charge from www.ccdc.cam.ac.uk/data_request/cif.

Table S1. Crystallographic data of **13**, **17**, **23** – **25**, and **30**.

Compound	**13**	**17**	**23**	**24**	**25**	**30**
Formula	$C_{12}H_{12}O$	$C_{24}H_{24}$	$C_{20}H_{18}$	$C_{22}H_{20}$	$C_{22}H_{20}$	$C_{18}H_{16}$
M_r	172.22	312.43	688.86	284.38	284.38	232.31
Habit	colourless prism	colourless tablet	colourless tablet	colourless lath	colourless tablet	colourless prism
Cryst. size (mm)	0.45 × 0.17 × 0.17	0.4 × 0.25 × 0.2	0.5 × 0.3 × 0.2	0.4 × 0.2 × 0.1	0.4 × 0.35 × 0.1	0.3 × 0.17 × 0.14
Crystal system	Monoclinic	orthorhombic	orthorhombic	monoclinic	monoclinic	monoclinic
Space group	$P2_1/n$	*Pbca*	*Pbca*	$P2_1/c$	$P2_1/c$	$P2_1/c$
Temperature (°C)	–140	–140	–100	–140	–140	–140
Cell constants:						
a (Å)	7.7201(16)	11.2648(12)	12.299(2)	6.2955(4)	17.526(3)	7.5017(8)
b (Å)	16.679(3)	8.9170(11)	8.1714(14)	17.6865(12)	12.9720(19)	4.8461(6)
c (Å)	14.454(3)	16.880(2)	14.361(2)	7.2886(5)	13.687(2)	16.923(2)
α (°)	90	90	90	90	90	90
β (°)	101.209(6)	90	90	111.585(1)	90.087(3)	94.615(4)
γ (°)	90	90	90	90	90	90
V (Å^3)	1825.7	1695.6	1443.3	754.64	3111.6	613.22
Z	8	4	4	2	8	2
D_x (Mg m^{-3})	1.253	1.224	1.189	1.252	1.214	1.258
μ (mm^{-1})	0.08	0.07	0.07	0.07	0.07	0.07
F(000)	736	672	552	304	1216	248
$2\theta_{max}$	56.6	60	56.6	60	52.7	60
Refl. measured	18234	12874	13549	8553	26177	6700
Refl. indep.	4525	2474	1789	2197	6358	1799
R_{int}	0.145	0.040	0.041	0.044	0.188	0.031
Parameters	239	111	91	100	398	82
Restraints	0	0	0	0	510	0
$wR(F^2$, all refl.)	0.163	0.142	0.115	0.125	0.219	0.123
$R(F, >4\sigma(F))$	0.064	0.050	0.041	0.046	0.090	0.043
S	0.98	1.04	1.08	1.03	1.06	1.07
max. Δρ (e Å^{-3})	0.33	0.40	0.22	0.46	0.72	0.43

References

1. Yildizhan, S. Kreuzkonjugierte π-Elektronensysteme-auf dem Weg zum Ur-Indigo. Ph.D. Thesis, Technical University Braunschweig, Germany, 2006. The present publication is based on the Ph. D. thesis of S. Yildizhan
2. Höpfner, Th.; Jones, P. G.; Ahrens, B.; Dix, I.; Ernst, L.; Hopf, H. *Eur. J. Org. Chem.* **2003**, 2596–2611. See for Part VIII doi:10.1002/ejoc.200300075
3. Ghosh, A. K.; Kincaid, J. F.; Haske, M. G. *Synthesis*, **1997**, 541-544.
4. Alcaraz, L.; Harnett, J. J.; Mioskowski, C.; Martel, J. P.; Le Grall, T.; Shin, D.-S.; Falck, I. R. *Tetrahedron Lett*. **1994**, *35*, 5449-5452.
5. Ensley, H. E.; Balakrishnan, P.; Hogan, C. *Tetrahedron Lett*. **1989**, *30*, 1625-1628 (yield: 49%).
6. Baierweck P.; Simmross, U.; Müllen, K. *Chem. Ber*. **1988**, *121*, 2195-2200.
7. Sheldrick, G. M. *Acta Cryst*. **2008**, *A64*, 112-122.
